# Informatics Approaches for Harmonized Intelligent Integration of Stem Cell Research

**DOI:** 10.2147/SCCAA.S237361

**Published:** 2020-01-28

**Authors:** Joseph Finkelstein, Irena Parvanova, Frederick Zhang

**Affiliations:** 1 Department of Population Health Science and Policy, Icahn School of Medicine at Mount Sinai, New York, NY, USA; 2 Center for Bioinformatics and Data Analytics, Columbia University, New York, NY, USA

**Keywords:** stem cells, data integration, databases

## Abstract

As biomedical data integration and analytics play an increasing role in the field of stem cell research, it becomes important to develop ways to standardize, aggregate, and share data among researchers. For this reason, many databases have been developed in recent years in an attempt to systematically warehouse data from different stem cell projects and experiments at the same time. However, these databases vary widely in their implementation and structure. The aim of this scoping review is to characterize the main features of available stem cell databases in order to identify specifications useful for implementation in future stem cell databases. We conducted a scoping review of peer-reviewed literature and online resources to identify and review available stem cell databases. To identify the relevant databases, we performed a PubMed search using relevant MeSH terms followed by a web search for databases which may not have an associated journal article. In total, we identified 16 databases to include in this review. The data elements reported in these databases represented a broad spectrum of parameters from basic socio-demographic variables to various cells characteristics, cell surface markers expression, and clinical trial results. Three broad sets of functional features that provide utility for future stem cell research and facilitate bioinformatics workflows were identified. These features consisted of the following: common data elements, data visualization and analysis tools, and biomedical ontologies for data integration. Stem cell bioinformatics is a quickly evolving field that generates a growing number of heterogeneous data sets. Further progress in the stem cell research may be greatly facilitated by development of applications for intelligent stem cell data aggregation, sharing and collaboration process.

## Introduction

Stem cells are defined as cells with the capacity for self-renewal and development into a specialized cell that composes healthy tissue.^1^ These cells were first described in 1961, when researchers James Till and Ernest McCulloch discovered the existence of self-renewing cell colonies in mice.^2^,^3^ The cells they discovered were later classified as hematopoietic stem cells, the first of many breakthroughs in the field of stem cell research.^3^ Since then, different types of stem cells have been discovered with the ability to differentiate into many different types of human tissue, including tissues that previously exhibited limited healing capacity such as neurons.^4^ The discovery of these cells has revolutionized the field of regenerative medicine, with many exciting potential applications for stem cell therapy in a variety of diseases and conditions previously thought to be incurable.

However, the field of stem cell studies is expensive and difficult to access for the majority of researchers. A major reason for this is the controversial nature of stem cell research and the ethical discussions which have ensued. Many of the developed nations in the world, including the United States and several European countries, have restrictive policies regarding stem cell research.^5^ The United States, in particular, has had an evolving history regarding the accessibility of stem cell research. Under the previous administrations, federal funding for research on new embryonic stem cell lines was halted, leading to a major slowdown in stem cell research in the US. This decision was later reversed under the next administration.^6^ The result is that the approaches to generate stem cells and use them in research are governed by a set of ethical and regulatory considerations. Under the current conditions, it is both expensive and challenging to create pluripotent stem cell lines for complex disorders.^7^

Part of the challenge in developing large numbers of stem cell lines is the difficulty in standardizing and optimizing stem cell differentiation protocols. In 2006, Kazutoshi Takahashi and Shinya Yamanaka discovered that pluripotent stem cells could be induced from fibroblasts through the expression of just four transcription factors.^8^ Since then, a number of different methods have evolved to induce pluripotency in cells. These methods involve alterations at multiple levels of cellular regulation.^9^,^10^ This ranges from DNA reprogramming factor delivery using viral or plasmid vectors, to mRNA or miRNA transfection, to direct delivery of proteins or other small molecule compounds.^10^–^13^ Although the development of this number of techniques has vastly improved stem cell differentiation efficiency, the eclectic and complicated nature of these techniques makes it difficult for these protocols to be disseminated between researchers. In order for a protocol to be successfully replicated, the researcher needs to have a detailed understanding of the biomarkers being expressed at each stage of differentiation as the gene expression of the final cell produced. For this reason, an important step in the progression of stem cell research is the development of stem cell data aggregates that collect detailed information regarding many stem cell lines into a single, easily accessible database. The potential amount of data being generated in each stem cell line analysis is immense. Providing centralized and easily accessible data repositories is necessary so that the data being generated can be fully utilized in stem cell research using various bioinformatics pipelines.

The creation of efficient and accessible data aggregates for stem cell research faces many challenges. In recent years, biological research has been revolutionized by the rise of the “OMICS”. The OMICS refers to a set of technologies which can provide a comprehensive understanding of the molecules and biomarkers present in a cell.^14^ This includes fields such as genomics, proteomics, and metabolomics, all of which contribute valuable information regarding the biological activity present in a cell at any given time. Naturally, OMICS techniques have played an integral role in the development of stem cell research. High throughput sequencing techniques have greatly enhanced researchers’ understanding of the gene regulation networks involved in establishing pluripotency in a cell.^15^ Accordingly, many of the challenges in data aggregation for stem cell research mirror the challenges in data aggregation of OMICS technologies. OMICS technologies generate massive amounts of data that often lack standardization and curation.^16^ Data aggregates which integrate OMICS datasets need to employ a standardized format for the data they collect and then develop a methodology for curating the data they choose to host. An additional significant challenge facing the integration of OMICS data into research is the need to relate raw data to a biological context.

The evolution and proliferation of OMICS technologies has generated data that has become increasingly granular and specific to the platform the data were collected on. This creates difficulty in integrating data across multiple datasets, particularly datasets using different platforms. In addition, it can be difficult to interpret these data in a biologic framework. For these reasons, the importance of using ontologies for biologic data aggregation has increased significantly in recent years. An ontology, broadly speaking, refers to a semantic method to categorize objects using relations and classes.^17^–^19^ The concept of using ontologies in biological sciences has been facilitated by the Gene Ontology project to categorize the role and function of genes.^18^,^19^ The Gene Ontology project was designed as a method to standardize the classification and annotations of genes across different research projects. Since the implementation of Gene Ontology, other ontologies for applications in biomedical research have been developed. The use of ontologies to standardize datasets and help researchers to find data and interpret the data they generate from a functional perspective is an important development for the advancement of stem cell research.^19^ For this reason, the integration of stem cell research into current ontologies is another important challenge facing stem cell database development.

Currently, a variety of databases exist which house information on different stem cell lines. However, these databases vary greatly in the data elements they capture, the search features they offer, and a number of other characteristics. Because of this, it is unclear what the best practices are in developing future stem cell databases. The field of stem cell research is rapidly growing, so naturally, the need for high-quality data storage and curation methods for stem cell data is growing alongside it. In this review, we evaluate both academic and commercial stem cell databases currently available. Our aim is to identify which useful features are available in high-quality stem cell databases, so that future stem cell databases can also incorporate these features for maximum utility and accessibility.

## Methods

For this paper, we conducted a scoping review of current stem cell data aggregates available. The scoping review methodology was established by Hilary Arksey and Lisa O’Malley^20^ as a method to quickly and broadly map out the up-to-date knowledge of a certain research area currently available.

### Scoping Review Workflow

The scoping review workflow generally follows five major steps:

1. Identify a research question
2. Identify relevant studies
3. Evaluate and select studies to be included
4. Chart the data
5. Collect, summarize, and report the results

### Research Question

Our goal in conducting this review was to identify the characteristics of the currently available platforms for stem cell researchers to aggregate and share data from multiple sources. The research question we aimed to address was “What are the characteristics of stem cell data aggregates and how can future databases improve upon these past iterations?”

### Search Strategy

First, we conducted a search on PubMed using the following MeSH term search: “Databases, Factual AND Stem Cells”. Because both stem cell research and data sharing are rapidly evolving fields, we chose 2010 as a publication cutoff year. We chose this year because, during our search, we found that many databases established prior to 2010 had been discontinued and the links contained within their respective papers led to un-hosted domains. These search terms resulted in 491 papers to be considered for inclusion in our final study.

We also conducted a web search for commercial stem cell databases that do not have associated papers indexed in PubMed.

### Study Selection

The following inclusion criteria for the PubMed articles were developed during our search process:

1. The paper must describe a stem cell experimental database.
2. The database must include human stem cell lines.
3. The database must include data extracted from stem cell lines which are retrievable.
4. The database must aggregate data from multiple datasets, as opposed to hosting only data from “in-house” cell lines.
5. Cancer stem cells databases are also included.
6. Databases that are no longer available were also included.
7. The database or related paper must be available in English.

The following exclusion criteria were then applied after the inclusion criteria:

1. Databases where a paper was published but the project itself was not completed.
2. Databases which include some stem cell data but stem cells are not the primary focus.

These same criteria were also applied to all databases found from the web search where applicable. After conducting our search and applying these criteria, we found 14 papers to include in our review from PubMed. Our web search found two additional databases to add to our study.

### Data Extraction

First, we obtained a general overview of the characteristics of each database as described by the authors, such as the primary goal of the project, the methodology for developing the database, the species included, data elements. Based on the results of the general overview, we developed seven major dimensions to capture the characteristics of each database. The dimensions are as follows:

1. Stored data elements: In this category, we described the data elements available for each individual dataset or data point within the database. For example, some databases provided a detailed information for each cell line alongside the raw data, including the tissue of origin, treatments performed, etc., whereas other databases may have only included the basic characteristics of a cell line such as the cell type and species.
2. Available data analysis tools, such as co-expression or covariate analysis: In this category, we included any supplemental tools provided in the database to perform analyses, which were not available from the original data (for instance, heatmap generation or cluster analysis).
3. Search features: In this category, we described how data could be searched for each database and any additional features implemented to refine or streamline the search process.
4. Storage platform and interface: In this category, we described the architecture used to host both the database itself and the user interface.
5. Privileges needed for access: For each database, we described the process for researchers to submit data as well as the privileges necessary to access the data, i.e. if the database was freely available, if it required registration, etc.
6. Update methodology: In this category, we described the current or planned methodology for periodic database updates.
7. Current status: In this category, we described the current status of the database and any updates or significant changes since the original paper was published.

In addition, we identified general features that could improve the utility of all future stem cell databases. The aim of most stem cell databases at their inception was to facilitate and streamline stem cell research. However, these databases greatly varied in the functionalities they implemented to reach this goal. We identified three important, broad database functional features amongst the reviewed databases that could be implemented in future stem cell research data repositories. They are as follows:

1. Data Elements: All available data elements were documented including sequencing data regarding specific stem cell lines such as microarray expression or Next Generation Sequencing (NGS) data. The primary aim of many databases is to aggregate large amounts of experimental data from multiple stem cell lines and trials. In the current landscape of stem cell research, traditional gene expression sequencing data and NGS data have emerged as the primary biomarkers for the understanding of stem cell induction and differentiation. Therefore, providing a direct source of information regarding these biomarkers is an important measurement of the utility of the database.
2. Data Analysis Tools: This includes data analysis tools supported by each database. Inclusion of these tools allows for a more streamlined integration of the available data for further analysis. Providing this feature makes stem cell informatics research across multiple datasets more accessible and feasible.
3. Ontology Integration: This includes integration with standard ontologies or self-developed nomenclatures to relate raw data to known biological frameworks. The natural overlap of the field of stem cell research with the “omics” (genomics, proteomics, etc.) means that it is often difficult to annotate the experimental data into a standardized and informative format. Mapping the experimental data elements to standard ontology concepts makes stem cell research more accessible to researchers across different disciplines, as well as improves the standardization of the integrated data. For this reason, ontology integration is another important feature to include to improve the utility of stem cell databases.

**Figure 1:**
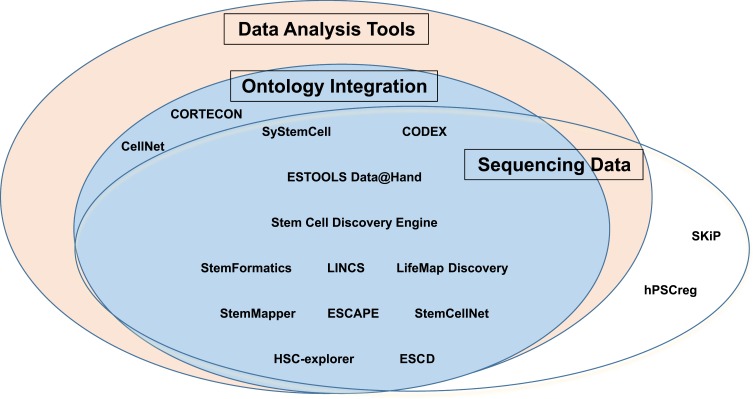
A diagram of major utility features in the reviewed databases.

After this identification, we inspected which of these features each database provided and generated an Euler diagram summarizing the functionality of reviewed databases in Figure 1. All database features were extracted using an iterative manual review. A detailed report of all data elements collected in the course of this review can be found in the Supplementary data.

## Results

### General Characteristics

In total, we found 14 academic databases from our PubMed search and two databases on the web which did not have an associated paper. These databases varied widely in the data elements, available data analysis tools, search functionality, and update methodology. The most common storage and interface architecture was data storage using a MySQL relational database with a web interface implemented on an Apache server, which was used by five databases. All of the databases were free to access; however, one required registration for full access. Three of the databases no longer had any data available. The oldest active database was the Embryonic Stem Cells Database (ESCD), which was published in 2010. Out of the 16 databases, LifeMap Discovery was the only one that included information about the stem cells applications in clinical trials. A detailed description of the data elements, used in each database can be found in Table 1. A report of the operational characteristics for each database has been outlined in Table 2.

**Table 1:** Data Elements in Stem Cell Databases

Database Tittle	Author	Data Elements	Link
SyStemCell	Yu et al^22^	Stem cell type and species; Gene annotations; DNA CpG 5 hmC/5 mC;	https://omictools.com/systemcell-tool
		Histone modification; Karyotype; Treatments performed on cell;	
		miRNA-based regulation; Protein abundance; Protein phosphorylation;	
Transcription factor regulation
CODEX	Sanchez-Castillo et al^23^	Next Generation Sequencing data; Cell type, subtype, and species;	http://codex.stemcells.cam.ac.uk/
		Tissue ontology; Cell culture conditions; Cell drug treatments;	
Human chromosomal abnormalities
ESTOOLS Data@Hand	Kong et al^24^	Microarray data; Sequencing platform; Species, sex, and disease status of donor; Tissue of origin; Cell type of parent and	https://research.utu.fi/converis/portal/Publication/1716702
current cell line; Differentiation status
Stem Cell Discovery Engine	Sui et al^25^	Microarray data; Species, strain, developmental stage, disease state of donor; Cell type and drug treatment; Tissue type and histology; Transcription profiling; Histone modification profiling;Transcription factor binding site identification; Immunoprecipitation antibody; Phenotype quality; Cell surface marker	http://discovery.hsci.harvard.edu/
StemFormatics	Wells et al^26^	Gene expression data; Sequencing platform; Cell type and subtype;	https://www.stemformatics.org/
Species, sex, age, and disease state of door; Tissue of origin
LINCS	Koleti et al^27^	Species, disease state, mutation status, and genetic modification status of donor; Tissue of origin; Cell culture conditions; Protein treatment information; Antibody treatment; Small molecule treatment information; siRNA/shRNA treatment information	http://lincsportal.ccs.miami.edu/dcic-portal/
LifeMap Discovery	Edgar et al^28^	Gene expression data; Anatomic compartment and tissue of origin; Cell type and subtype; Cell culture conditions and treatments; Related clinical trials for cell therapies	https://discovery.lifemapsc.com/
StemMapper	Pinto et al^29^	Microarray data; Species, age, and mutation status of donor; Cell type and subtype	http://stemmapper.sysbiolab.eu/
ESCAPE	Xu et al^30^	Cell surface markers; mRNA expression; Protein-protein interactions;	http://www.maayanlab.net/ESCAPE/index.php
Chip-Seq interactions; miRNA target interactions; Histone modification; Gene annotations; Cell type
SKiP Stemcell Knowledge and Information Portal		Species, age, sex, disease state, and ethnicity of donor; Tissue of origin;	https://skip.stemcellinformatics.org/en/
Cell type; Cell morphology; Culture medium, feeders; Karyotype
hPSCreg		Sex and disease state of donor; Cell type; Cell derivation process;	https://hpscreg.eu/
Cell culture conditions; Cell surface markers
CellNet	Cahan et al^31^	Stem cell subtype; Stem cell species; Sex of the donor; Transcription factors;	http://cahanlab.org/macellnet.html
Microarray data
StemCellNet	Pinto et al^32^	Stem cell type; Stem cell lineage; Stem cell species; Cell surface markers;	http://stemcellnet.sysbiolab.eu/
		Transcription factors; Immunoprecipitation antibody; Microarray data;	
Sequencing platform
HSC-explorer	Montrone et al^33^	Stem cell lineage; Stem cell species; Differentiation status; Cell surface markers; Gene expression data; mRNA expression; Transcription profiling;	http://mips.helmholtz-muenchen.de/HSC/
Sequencing platform
CORTECON	van de Leemput et al^34^	Stem cell type; Stem cell lineage; Stem cell species; Disease state of the donor; Cell surface markers; Gene expression data	http://cortecon.neuralsci.org/
ESCD	Jung et al^35^	Stem cell type; Stem cell lineage; Stem cell species; Cell surface markers;	https://biit.cs.ut.ee/escd/
		Transcription factors; Immunoprecipitation antibody; Gene expression data;	
Sequencing platform

**Table 2:** Operational Characteristics of Stem Cell Databases

Database	Search Features	Storage Platform and Interface	Privileges Needed for Access	Update Methodology	Current Status
SyStemCell	- Browse by organism, level of regulation, stem cell type, or control sample	- Stored in MySQL relational database and configured on RedHat Linux Server	Free access	Not specified	No longer available
	- Search by Gene Entrez ID, gene symbol, or gene alias	- Web interface implemented on an Apache server			
	- Online analysis tools developed in R
CODEX	- Browse by cell type, repository, transcription factor, histones and associated proteins, or sequencing platform	- Not specified	Free access	Self-developed web crawler which constantly searches GEO database	Still available, unclear if still being updated
	- Search by transcription factor, gene, or cell type
ESTOOLS Data@Hand	- Can search by any annotation parameter: organism, cell type, disease state, etc.	- Stored in MySQL relational database	Free access with registration	Manually updated four times per year	No longer available
- Web interface implemented on Apache server
Stem Cell Discovery Engine	- Search by free text, organism, measurement, technology, or platform	- Implemented on Harvard’s custom Galaxy server system	Free with registration	Researchers submit experiments which are then manually curated	Still available but has been merged into Harvard’s Stem Cell Commons data repository
StemFormatics	- Search by gene, species, author, platform, or cell type	- Not specified	Free access but registration needed to access certain features	Manually updated	Still active and available
LINCS	- Search by datasets, small molecules, or cells via any annotation	- Stored in PostgreSQL database	Free access	Data is submitted and uploaded via the LINCS Data Registry protocol	Still active but all features not yet available
	- Free text search of all data with Boolean logic integration	- Web interface implemented using Apache Solr		
- Search by chemical structure by uploading mol files			
LifeMap Discovery	- Search by organ/tissue, cell type, related cell therapies, or gene expression	- Not specified	Free access but registration needed for access to all features	Not specified	Still active and available
StemMapper	- Search by tissue, cell type, and gene	- Stored in MySQL relational database	Free access	Not specified	Still active and available
- Web interface implemented using JavaScript ad JavaServer Faces 2.1
ESCAPE	- Browse by data type	- Stored in MySQL relational database	Free access	Not specified	Still available
	- Search by gene name, gene ID, cell type, PubMed ID, or submission date	- Displayed via Cytoscape web plugin			
	- Web interface implemented on Apache server and Java server
SKiP Stem Cell Knowledge and Information Portal	- Search by associated disease, cell type, tissue type, disease status, donor characteristics, vector, database, PubMed ID, or text search	Not specified	Free access	Not specified	Still available but last updated in 2017
hPSCreg	- Search by country, date, PubMedID, disease, cell type, or free text search	Not specified	Free access but registration needed for access to all features	Not specified	Still active and available
CellNet	- Browse by organism	- Online analysis tool developer in R	Free access	Not specified	Computational platform is available
	- Search by cell deriviation, transcriptional targets, gene regulatory	- Affy packages by Bioconductor need to be installed			
-Review targets, gene regulatory network of starting cell type, gene expression	- Web interface implemented using JavaScript and JavaServer Faces 2.1
StemCellNet	- Browse by organism	- Not specified	Free access	Not specified	Still available versitile platform
- Search by proteins, transcription factors, genes
HSC-explorer	- Browse by Gene, protein, SNP, biological process, cellular component, tissue, cell line, organism, chemical compound	- Not specified	Free access	Manually curated	The platform is expanded to cover all hematopoietic progenitors
‘- Search by entry ID, Pubmed ID, comment, author, etc.
CORTECON	- View by gene, disease, KEGG pathway, GO ontology	- Not specified	Free access	Not specified	Available for download
- Provides tools for browsing genes, expressed during cortical development
ESCD	- Browse by Ensembl gene IDs and gene names				
- Browse by organism, transcription factors, GO terminology	- Not specified	Free access	Not specified	Datasets originate from genome version platforms

With regard to major database features, the majority of the academic databases contained sequencing information but did not provide access to the sequencing data. Of all included databases, 14 provided data analysis tools alongside the sequencing information (Table 3). In 14 databases, standard ontology concepts or specifically developed terminologies were integrated with raw data stored in the database and used for further data representation and analysis (Table 4). In total, 12 of the databases we reviewed had all three functional features.

**Table 3:** Data Analysis Tools Used in Stem Cell Databases

Database	Data Analysis Tools
1. SyStemCell	1.1 Co-localization analysis; 1.2 Venn Diagram plotting; 1.3 DAVID enrichment analysis
2. CODEX	2.1 Motif discovery analysis and peak profile correlation analysis
3. ESTOOLS Data@Hand	3.1 Differential expression analysis; 3.2 Co-expression analysis; 3.3 Clustering and heatmap analysis; 3.4 Gene expression profiling; 3.5 Sample-wise clustering and dendrogram generation; 3.6 GO and KEGG enrichment
4. Stem Cell Discovery Engine	4.1 Gene list comparison by gene signatures, molecular signatures, and pathways
5. StemFormatics	5.1 Gene clustering and heatmap analysis through Hamlet; 5.2 Hierarchical clustering and comparative marker selection through GenePattern module
6. LINCS	6.1 Drug pathway browser; 6.2 LINCS analytics; 6.3 Drug/Cell line Browser; 6.4 Repurposing App; etc.
7. LifeMap Discovery	7.1 Partnered with custom GeneAnalytics gene analysis suite
8. StemMapper	8.1 Heatmap, Principal Components Analysis, and Pearson correlation analysis
9. ESCAPE	9.1 Enrichment analysis and lineage prediction
10. SKiP Stem Cell Knowledge and Information Portal	- None
11. hPSCreg	- None
12. CellNet	12.1 R software analysis of protein and transcription regulatory interactions
13. StemCellNet	13.1 Web-based analysis of molecular networks; 13.2 Statistical analysis examining physical protein interaction, transcriptional reg. interactions;
13.3 Online statistical analysis platform allowing up to 500 genes input
14. HSC-explorer	14.1 Bioinformatics resource allowing the search of pathways correlated to hematopoiesis;
	14.2 Web-based analysis platform allowing visualization of data. Diagrams can be downloaded as SBML, graphML, or jpg files;
14.3 Database is linked to EntrezGene, KEGG, miRBAse, Gene Ontology, or CORUM
15. CORTECON	15.1 R-analysis using developing cerebral cortex transcriptome in humans; 15.2 Online resource allowing multiple views of developing cortex data
16. ESCD	16.1 Online repository which contains perturbation and ChIP experiments in ESCs

**Table 4:** Ontology-Based Applications in Stem Cell Databases

Databases	Ontology Interactions
1. SyStemCell	1.1 Gene Ontology database; 1.2 Biocarta Pathway; 1.3 Biosystems Pathway, and 1.4 dbDEPC database (correlation analysis).
2. CODEX	2.1 Gene Quest (TF’s targets and gene associations for human samples; ChIPpeakAnno and peak-to-gene associations).
3. ESTOOLS Data@Hand	3.1 Differential expression analysis; 3.2 Co-expression analysis; 3.3 Clustering and heatmap analysis; 3.4 Gene expression profiling.
3.5 Sample-wise clustering and dendrogram generation; 3.6 GO and KEGG enrichment.
4. Stem Cell Discovery Engine	4.1 Galaxy analysis software to enhance ontology searches.
5. StemFormatics	5.1 Gene clustering; 5.2 Hierarchical clustering and comparative marker selection through GenePattern module
6. LINCS	6.1 Drug-pathway browser, TieDIE (HMS LINCS); 6.2 Drug/Cell line Browser, Enrichr, Drug Response Browser (BD2K-LINCS DCIC);
6.3 Omics Integrator (Neuro LINCS); 6.4 ICV App (LINCS Transcriptomics), etc.
7. LifeMap Discovery	7.1 A detailed description of the developmental ontology of organ/tissues, anatomical compartments and cells; 7.2 Gene expression profiling in adult mammalian organs, tissues, anatomical compartments and cells, cultured stem, progenitor and primary cells, or cells derived via differentiation protocols to allow characterization of cells by gene expression patterns.
8. StemMapper	8.1 Heatmaps; 8.2. Correlation analysis.
9. ESCAPE	9.1 GOI enrichment with ESCAPE-listed genes RNAi screens, protein lists from IP-MS pull-downs, genes differentially expressed after knock-down or over-expression, and target genes for transcription factors and histone modifications as determined by ChIP-seq.
10. SKiP Stem Cell Knowledge and Information Portal	n/a
11. hPSCreg	n/a
12. CellNet	12.1 Analysis of protein and transcription regulatory interactions.
13. StemCellNet	13.1 Statistical analysis examining physical protein interaction, transcriptional reg. interactions; 13.2 Online statistical analysis platform allowing up to 500 genes input.
14. HSC-explorer	14.3 Database is linked to EntrezGene, KEGG, miRBAse, Gene Ontology, or CORUM.
15. CORTECON	15.1 Analysis using developing cerebral cortex transcriptome in humans.
16. ESCD	16.1 Query by Gene ID; 16.2 Query by GO term.

Overall, all 16 databases included 35 rubrics that characterized these databases (Tables 5 and 6). In Table 6, we listed all rubrics, used to describe stem cell populations, which included characteristics, such as stem cell line names, lineage, cell surface markers, transcription factors, and epigenetic modifications, as well as types of detection assays, data analysis tools, and clinical trials information. Due to the variety of rubrics characterizing stem cells databases, we used minimum information about a cellular assay for regenerative medicine (MIACARM) five-module classification system^21^ to further group and organize the 35 rubrics under the five MIACARM modules: project, assay, source cell, experimental technology, and data modules (Table 6).

**Table 5:** Stem Cell Databases are Characterized by Different Number of Rubrics

Stem Cells Databases	# of Rubrics per Database
SCDE	21
CODEX	16
ESTOOLS	16
SyStemCell	15
StemFormatics	14
StemMapper	14
SKiP Stemcell	13
LifeMap Discovery	12
ESCAPE	12
hPSCreg	12
StemCellNet	12
HSC-explorer	12
ESCD	12
LINCS	10
CORTECON	10
CellNet	9

**Table 6:** Representation of Characterization Rubrics in Stem Cell Databases (Plus Sign Indicates Presence in a Database)

MIACARM Module	Rubric	SyStemCell	CODEX	ESTOOLS	SCDE	StemFormatics	LINCS	LifeMap Discovery	StemMapper	ESCAPE	SKiP	hPSCreg	CellNet	StemCellNet	HSC-explorer	CORTECON	ESCD
1. Project	1. Summary	+	+	+	+	+	+	+	+	+	+	+	+	+	+	+	+
	2. Organization	+	+	+	+	+	+		+		+	+			+	+	+
	3. PI/Corresponding researcher			+	+	+			+				+	+			
4. Publication	+	+	+	+	+	+	+	+	+			+	+	+	+	+
2. Assay	5. Clinical Trials for Cell Therapies							+									
3. Source Cell	6. Stem Cell Type/Name	+	+	+	+	+		+	+	+	+	+	+	+		+	+
	7. Stem Cell Subtype		+			+		+	+								
	8. Stem Cell Lineage	+	+	+	+	+	+	+	+	+	+	+	+	+	+	+	+
	9. Stem Cell Species	+	+	+	+	+	+	+	+	+	+	+	+	+	+	+	+
	10. Sex of the Donor			+							+	+					
	11. Age of Donor					+			+		+						
	12. Ethnicity of the Donor										+						
	13. Disease State of Donor			+	+	+	+				+	+			+	+	
	14. Developmental Stage				+												
	15. Cell Derivation Process											+					
	16. Differentiation Status			+								+			+		
	17. Cell Morphology										+						
	18. Phenotype Quality				+												
	19. Tissue Type		+	+	+	+	+	+			+						
	20. Tissue Histology				+												
	21. Cell Surface Markers	+			+				+	+		+		+	+	+	+
	22. Transcription Factors Expression	+	+		+								+	+			+
	23. Epigenetic Modification	+	+		+					+							
	24. Gene Annotation	+								+							
	25. miRNA-based Regulation/Interactions	+								+							
	26. Protein Phosphorylation	+															
	27. Karyotype			+							+	+					
	28. Mutation Status of a Donor		+				+		+								
29. Cell Culture Conditions		+	+	+		+	+			+	+					
4. Experimental Technology	30. Immunoprecipitation Antibody				+									+			+
	31. Gene Expression Data					+		+							+	+	+
	32. Microarray Data		+	+	+				+				+	+			
33. miRNA Expression/Transcription	+	+		+					+					+		
5. Data	34. Sequencing Platform	+	+	+	+	+	+	+	+	+				+	+		+
35. Data analysis tools	+	+	+	+	+		+	+	+			+	+	+	+	+

Additionally, in Table 7, we examined in further details the complexity of each of the 35 rubrics with complexity expressed as the breadth of possible options which were used across the reviewed databases for a particular rubric. For instance, for “Cell Lineage”, we listed eight different stem cell types: adult stem/progenitor cells; embryonic progenitor cells; embryonic stem cells (ESC); fetal stem/progenitor cells; induced pluripotent stem cells (iPSC); cell lines; primary cells, and tissue/cells composite. On the other hand, a rubric, such as “Transcription Factors Expression”, features 210 transcription factors just in the CODEX database. Other rubrics, such as Cell “Surface Markers”, include a large number of proteins, which are too diverse to be accurately counted. To complete this portion of the analysis, we represented the frequency of the usage of each rubric for characterizing a stem cell database by calculating the percentage of the appearance of that particular rubric in all 16 databases.

**Table 7:** Complexity of Rubrics Characterizing Stem Cell Research Databases

Stem Cells Database Rubrics	Complexity of Each Rubric	Complexity Breakdown	% of Databases with the Rubric
Summary	N/A	N/A	100%
Stem Cell Lineage	8	Adult stem/progenitor cell; embryonic progenitor cell; ESC; fetal stem/progenitor cell; iPSC; cell lines; primary cells; and tissue/cell composite	100%
Stem Cell Species	5	human, mouse, rat, pig, macac	100%
Publications	N/A	N/A	88%
Stem Cell Type/Name	Thousands of stem cell types		88%
Data analysis tools	28	Co-localization analysis; Venn Diagram plotting; DAVID-enriched analysis; peak profile correlation analysis; gene set control analysis; motif discovery analysis; differential expression analysis; clustering and heatmap analysis; gene expression; GO and KEGG enrichment; gene list comparison, and 15 more	88%
Organization	N/A	N/A	75%
Sequencing Platform	Next Generation Sequencing	Illumina, others	75%
Cell Surface Markers	Large number of cell surface proteins		56%
Disease State of Donor	Large number of diseases	X-linked juvenile retinoschisis; Pathological myopia; Short rib-thoracic dysplasia syndrome; DFNB4,	50%
		Pendred syndrome; Xp22.2 Exon; Childhood acute B-lymphoblastic leukemia; Parkinson’s disease;	
Alzheimer’s disease; Fragile X-syndrome and others
Tissue Type	Large number of tissue types	Blood; Adipose; Mesenchymal Stem Cells; Endothelium; Bone; Heart; Cartlidge; Liver; Amnion;	44%
Tooth; Placenta; Uterus; Epithelial Cells; Umbilical Cord, and others
Cell Culture Conditions and Treatment	Variety of conditions		44%
PI/Corresponding Researcher	N/A	N/A	38%
Transcription Factors Expression/Regulation	210 (CODEX); +9 other databases	RUNX1; CBFB; CEBPA; CTDP1; EP300; ERG; FUSELF1; ERG; FLI1; GATA2; HDAC1; MYH11	38%
RUNX3; CDK7; CDK9; POLR2A; BCL6; BCOR; SMRT; ZNF143; SPI1; BACH2, and others
Microarray Data	420 microarray datasets (StemFormatics); +5 other databases		38%
Gene Expression Data	10,401 (LifeMap Discovery); 5813 (ESCAPE); +4 other databases		31%
miRNA Expression/Transcription Profiling	N/A	N/A	31%
Stem Cell Subtype	132 (CODEX); +3 other databases	[CL]CMK; [PC]CD34+ acute myeloid leukemia blast cells; [CL]Kasumi-1+ scrambled siRNA;	25%
		[CL]ME-1; [CL]NB4; [CL]Lymphoblastoid; [PC] H1 ES derived angiogenic hematopoietic progenitor;	
[PC] H7 ES derived anterior foregut, and others
Epigenetic modifications	72 (CODEX); +11 other databases (Histone Modifications)	H3K4me1; H2A.Zac; H3K9Ac; H3K9K14ac; H2AFZ/H2A.Z; H3K4me3; H4K20me3; H3K27ac;	25%
H3K27me3; H3K4me2; Ab4729; H3K79me2; H3Y41ph, and others
Sex of the donor	2	Female, male	19%
Age of the Donor	N/A	N/A	19%
Differentiation Status	N/A	N/A	19%
Karyotype	N/A	N/A	19%
Mutation Status of a Donor	Large number of abnormalities listed (CODEX); +2 other databases	Trisomy 21; translocation t(16,21; Acute Monocytic Leukemia; cervical adenocarcinoma;	19%
p53 mutation; K562 erythrocytic leukaemia cells; erythrocytic leukemia cells; and others
Immunoprecipitation Antibody	N/A	N/A	19%
Gene Annotation	N/A	N/A	13%
miRNA-based Regulation/Interactions	N/A	N/A	13%
Clinical Trials for Cell Therapies	568 (LifeMap Discovery)		6%
Ethnicity of the Donor	N/A	N/A	6%
Developmental Stage	N/A	N/A	6%
Cell Derivation Process	N/A	N/A	6%
Cell Morphology	N/A	N/A	6%
Phenotype Quality	N/A	N/A	6%
Tissue Histology	N/A	N/A	6%
Protein Phosphorylation	N/A	N/A	6%

#### SyStemCell^22^

SyStemCell is a database that aims to collect stem cell data at seven different levels of regulation: DNA CpG methylation, histone modification, transcript products, microRNA-based regulation, protein products, phosphorylation proteins, and transcription factor regulation. Stem cell data from four species are included: *Homo sapiens, Mas musculus, Rattus norwegicus*, and *Macaca mulatta*. Users can search by a specific gene or browse by organism, tissue type, etc. SyStemCell also offers Co-Localization analysis tools to allow researchers to investigate correlations between various genes at their different levels of regulation. Currently, the web domain for SyStemCell is no longer functional and the tool no longer seems to be available.

#### Codex^23^

CODEX is a database that aims to collect data from Next Generation Sequencing (NGS) experiments and condense them into an easily searchable, curated database. Formats supported by CODEX include ChIP-Seq, RNA-Seq, and DNase-Seq. The primary purpose of CODEX is to provide NGS data for hematopoietic and embryonic stem cells in a standardized format. All data uploaded to CODEX has gone through a bioinformatics pipeline to be processed into a standardized format and curated for all relevant details. CODEX contains data from both human and mouse samples. Users can browse and search by both cell type or transcription factor. CODEX offers a number of different gene analysis tools, such as correlation analysis and motif analysis. Currently, CODEX is available online and being actively updated.

#### ESTOOLS Data@Hand^24^

ESTOOLS Data@Hand is a database that aims to allow users to browse gene expression data from published stem cell research. The database was generated from manually selected experiments from Gene Expression Omnibus (GEO) and ArrayExpress which were then manually curated into a standardized format. The data can be browsed or searched by any annotation dimension. The database offers a set of analysis tools based on the Bioconductor package in R, such as co-expression and clustering tools. The authors also created two meta-datasets, one from combining all Affymetrix datasets and the other from combing all Illumina datasets. Currently, the web domain for ESTOOLS Data@Hand is no longer functional and the database no longer seems to be available.

#### Stem Cell Discovery Engine^25^

The Stem Cell Discovery Engine is a database designed to integrate data from both tissue stem cells and cancer stem cells experiments. Data from these experiments are both collected from public studies as well as user-submitted and manually curated for relevant data. Metadata from the experiments are stored in the Investigation/Study/Assay format. Genes selected in the database can be queried against other gene annotation databases for related genes or gene annotation information. The Stem Cell Discovery Engine also offers features to share gene sets in order to facilitate collaboration with other researchers. Currently, the Stem Cell Discovery Engine is active and being updated; however, it has been merged into Harvard’s larger Stem Cell Commons project.

#### StemFormatics^26^

StemFormatics is a database of public human stem cell datasets manually collected and curated from public sources such as GEO or ArrayExpress. Microarray datasets are subjected to a quality control bioinformatics pipeline before inclusion into the database. StemFormatics allows users to query by gene name or identifier and view gene expression data from a single gene across multiple datasets simultaneously. Clustering and heatmap generation tools are also available as gene analysis tools. The database also provides a Workbench feature that allows users to create and share gene sets for collaborative analysis. Currently, the StemFormatics database is active and being updated.

#### LINCS^27^

LINCS is a database designed to capture data from cell-perturbation and response experiments. This includes a large number of stem cell lines reflecting the number of experiments dedicated to discovering the conditions to differentiate and maintain stem cells. LINCS processes data through its associated LINCS Data Registry, which standardizes and curates the data and metadata. These data include information on cell culturing conditions, reagents and perturbation agents used to induce cell responses, such as small proteins and immunoglobulins, and the resulting cell line biomarkers. The LINCS database includes many of the traditional “browse and search” features shared with the other databases, as well as the unique ability to search for projects involving a specific molecule or similar molecule by uploading the mol file.

#### LifeMap Discovery^28^

LifeMap Discovery is a comprehensive stem cell database that aims to share stem cell data with researchers along with potential applications to regenerative medicine. The LifeMap Discovery webpage is unique in providing its own ontology tree of the current understanding of embryonic and stem cells differentiation to facilitate stem cell research. This ontology tree also contains reference data and genes which are relevant to that specific cell or tissue type. In addition to its ontology tree, LifeMap Discovery also contains a database of stem cell experiments, which can be browsed by organism, tissue type, gene, and other ontological characteristics. Users can also browse experiments by their application to stem cell and regenerative medicine therapies. The LifeMap Discovery database is partnered with the GeneQuest data analysis suite, which provides its own set of gene analysis features. Currently, the LifeMap Discovery database is active and still being updated.

#### StemMapper^29^

StemMapper is a database of gene expression data for both human and mouse stem cell lines manually collected and curated from GEO. StemMapper contains only data from experiments using certain Affymetrix sequencing platforms. Users can query the database by gene, as well as browse by cell and tissue type. Heatmap generation and Principal Components Analysis are available as built-in data analysis tools. In addition, StemMapper offers a unique feature for users to upload their own gene expression datasets for analysis. The StemMapper database is still available but has not been updated since 2017.

#### ESCAPE^30^

ESCAPE is a stem cell database which aims to provide stem cell experiment data at multiple levels of regulation, such as epigenetics, transcriptomics, and proteomics. Using ChIP-seq data, the ESCAPE database localizes transcription factors to their genes, characterizes protein/DNA and other protein interactions, among other biomarkers. The database can be searched by cell type, gene name, or platform, among others, or can be browsed by the level of regulation. ESCAPE offers enrichment analysis and a lineage prediction tool as its data analysis features. Currently, the ESCAPE database is available but is not being updated.

#### SKiP Stemcell Knowledge and Information Portal

SKiP is a stem cell experiment database developed and operated in Japan. The database contains information on stem cell lines which can be searched by characteristics, such as tissue type, cell type, as well as by their potential applications to disease therapy. Currently, SKiP is still available but no longer actively updated.

#### hPSCreg

hPSCreg is a stem cell experiment registry based in Europe which contains information on stem cell lines and experiments. The database allows users to submit and edit their own stem cell line data as well as requisition access to other stem cell lines. The database can be searched by free text, country of origin, or associated disease. Currently, hPSCreg is active and still being updated.

#### CellNet^31^

CellNet database was an online tool, which allowed microarray data of stem cell populations to be uploaded and evaluated. Currently, the database is no longer available; however, its code could be utilized for the microarray data analysis. The output of the analysis provides information about the cell species, lineage, tissue type, and transcription factors expression.

#### StemCellNet^32^

StemCellNet is a database, which includes data about (1) physical protein interactions; (2) transcription regulatory interactions, detected by chromatin immunoprecipitation, combined with microarray and sequencing technologies; and (3) gene interactions, which focuses on the genes that define the “stemnesses” of the stem cell lines. The platform allows for the assessment and visualization of the molecular networks. The tool allows as many as 500 genes to be simultaneously analyzed. The platform is versatile, free to access, and still available.

#### HSC-Explorer^33^

HSC-explorer allows access to information on the early differentiation stages of hematopoietic stem cells. It provides information about the species, molecular interactions and signaling process in these cells, as well as the performed assays. The data can be displayed as graphical representation, allowing web-based analysis, where the hematopoietic stem cell repository is publicly available and manually updated.

#### Cortecon^34^

CORTECON is a repository of transcriptome analysis of genes in human embryonic stem cells, expressed in the developing human cortex. The transcriptome analysis can be viewed by gene, disease, KEGG pathway, and enriched ontologies. The data can be analyzed using R-software. The database is freely accessible and still updated.

#### Embryonic Stem Cell Database (ESCD)^35^

ESCD contains mouse and human embryonic stem cell line information from embryonic stem and carcinoma cells. The included datasets cover perturbation (both knock-down and overexpression) and chromatin immunoprecipitation (ChIP) experiments in stem cell lines. Transcription factor binding data originate from various ChIP experiments (ChIP-PET, ChIP-chip, ChIP-seq). The database enables gene-based queries and supports the search for genes with a specific behavior in selected datasets. Datasets obtained from various array and genome version platforms are linked using Ensembl gene IDs and gene names. The database is publicly accessible and still available.

## Discussion

Our review identified 16 databases created since 2010 which aggregate data on stem cell projects. Of those databases, three are no longer available and several others appear to have stopped updating. When we examined the data elements being tracked by each database, we found a great deal of heterogeneity. Some databases warehoused curated and formatted genotype information or next-generation sequencing data, whereas others only provided information regarding the cell line itself. The databases had their own methods of curation and formatting of experimental and clinical data which varied significantly between each database. Many of the databases had similar search functions but differed in the ways they implemented them and in the user-friendliness of their interface. Almost all of the databases were freely available to browse without registration, with the exception of the Stem Cell Discovery Engine, which required registration to access data.

Many of these databases had similar aims: to provide a centralized source of data for stem cell experiments where the data are housed in a standardized, curated, and searchable format. The hope is that in doing so, it becomes easier for researchers to analyze data across multiple data sets. In order to facilitate this goal, we identified three broad features which could be implemented in future stem cell data aggregates to improve their accessibility and utility for researchers. Housing curated sequencing data and providing data analysis tools within the database itself are features which allow the data to be easily integrated into a bioinformatics workflow. Integration of the database with established biomedical ontologies allows the analyzed data to be interpreted in a more standardized format between different researchers, particularly in fields in which our understanding of certain data elements is still rapidly evolving, such as the OMICS. Understanding the database features which improve the stem cell research workflow and collaboration process is important in advancing stem cell research and utilizing the vast amount of aggregated data for further knowledge discovery.

The results of this study reflect a systematic abstraction of all data elements and functional features of reviewed stem cell databases to provide detailed insights for future improvements in accessibility and utility. The current literature examining the characteristics and development of stem cell databases is fairly limited.^36^,^37^ A review of stem cell databases was also published in 2015.^38^ This review provides only summaries of the important features as well as subjective rating score for each database. A more general review of all biological databases was reported in 2015.^39^ This review provides a broad overview of biological databases covering many different fields in biomedical research and categorizes them based on characteristics such as their scope of coverage and method of curation. However, because the characteristics of databases in different fields of biomedical research serve different purposes, and therefore require different features, the perspectives from this study are difficult to translate to a relatively new and evolving field of stem cell research. Our review differs from previously published work in several aspects. First, a systematic study design based on a scoping review methodology has been employed. Second, we chose to evaluate the individual features of each database, such as their data element characterizations, availability of analytical pipelines, and ontology integration, rather than provide an overall evaluation of each database. This allowed identify valuable and unique features from each database which can be integrated into future databases. Third, we used MIACARM as the current framework for stem cell research data reporting to characterize the scope of the existing databases. Therefore, this review provides new systematic information that potentially can be instrumental in improving the stem cell research analytical databases.

Stem cell studies have become such a highly investigated field in recent years because of their potential for developing new therapies for difficult to treat diseases. For certain conditions, stem cell therapy has already been available for some time. The use of hematopoietic stem cell transplants has been established as an effective therapy for hematologic malignancies such as leukemia and multiple myeloma.^40^ Because of the success of these treatments as well as the theoretically limitless growth potential of stem cells, stem cells have become the new frontier for the treatment of a variety of chronic conditions.^41^,^42^ For example, stem cells are now being investigated for their potential to treat degenerative diseases, such as multiple sclerosis,^43^ Parkinson’s disease^44^ or myocardial ischemia.^45^ Despite the fact that many theoretical models for these treatments have been developed, none have yet been translated into routine clinical care. For this reason, a comprehensive and systematic aggregation of current and future results of preclinical studies and stem cell therapeutic interventions is crucial for advancing this field towards practical implementation. Our review identified a number of stem cell databases that stopped updating their content or were not accessible anymore at all. This finding demonstrated the importance of establishing a sustainable framework for continuing and reliable maintenance of stem cell databases. Thus, in addition to effective informatics solutions to aggregate data across different repositories, investment also needs to be made in sustaining these repositories including financial support for the infrastructure and suitable incentives and career recognition for data curators working to update, clean and maintain these databases and keep them available for open access.

Although this scoping review was intended to be comprehensive, several stem cell databases did not satisfy inclusion/exclusion criteria or were not included due to the limitations of the search criteria. There may be additional stem cell databases which may not necessarily have an associated peer-reviewed article or require different keywords for successful web search. Such repositories of stem cell resources as the European Bank for induced pluripotent Stem Cells (EBiSC)^46^ and RIKEN BioResource Center^47^ may serve as important additional resources for stem cell data. Since we characterized all reviewed databases in a granular and systematic way and many features were present in at least a subset of the 16 reviewed databases, an omission of a few databases is unlikely to result in loss of substantive features. Overall, this review resulted in a comprehensive and detailed analysis of stem cell databases currently available.

Recognizing the importance of establishing an effective ecosystem for data aggregation and sharing, a diverse set of stakeholders representing academia, industry, funding agencies, and scholarly publishers have recently introduced principles for scientific data management and stewardship to improve the findability, accessibility, interoperability, and reuse (FAIR) of digital assets.^48^ The FAIR guiding principles for research data stewardship could provide a necessary framework for the future development of stem cell databases. Recent publications reported successful approaches for aggregating multiple heterogeneous data streams representing diverse molecular signatures of various cell types in a harmonized and expandable way. For example, the Library of Integrated Network-based Cellular Signatures (LINCS) project, a multi-center NIH-funded program, created a comprehensive library of molecular signatures supporting data integration, modeling and analysis methodologies.^49^ Broad sharing of genomic- and health-related data requires proper governance and security.^50^ In the context of stem cell research, data and sample sharing represent a scientific and ethical challenge to ensure appropriate protection of individual interests as well as maintaining public trust. Effective data protection requirements are necessary along with the future data harmonization efforts for building successful stem cell research data sharing.^51^ Deployment of the common framework for responsible sharing of genomic and health-related data established by the Global Alliance for Genomics and Health (GA4GH) in stem cell databases can facilitate the use of data in compliance with national and international laws and general ethical principles and standards.^52^

Future informatics approaches for harmonized intelligent integration of stem cell research data are dependent on timely introduction of a spectrum of standards concerning informed consent both from donors and recipients, procurement of biomaterials, manufacturing regulations, cell potency assays, minimally acceptable changes during cell culture, methods of recipient identification for experimental interventions, reporting of preclinical experiments, clinical trial design and reporting, and principles for defining common data elements in stem cell datasets.^53^ The Guidelines for Stem Cell Research and Clinical Translation established by the International Society for Stem Cell Research (ISSCR) in 2016 provide a comprehensive set of principles for rigor, oversight and transparency in all aspects of stem cell science and practice.^54^ The International Stem Cell Banking Initiative (ISCBI) promulgates best practices for manufacture, culture, characterization, storage, distribution, and translation of stem cell products.^55^ For example, ISCBI has taken the lead in defining critical quality attributes for induced pluripotent stem cells (iPSC) and issued recommendations on the minimum dataset required to consider an iPSC line of clinical grade which includes such attributes as identity, microbiological sterility, endotoxin, genetic fidelity & stability, variability, characterization and potency.^56^ Another example of standardized evaluation of stem cell-based products is the recently proposed multiparametric quality assessment rubric for stem-cell derived cardiac myocytes.^57^

To catalyze knowledge discovery in stem cell research, the scientific community needs to adopt intelligent technologies that harmonize multiple heterogeneous data streams utilizing internationally accepted standards. Policies that reward approaches supporting interoperability and promoting the expansion of open data commons will facilitate findability, accessibility, interoperability, and reuse of stem cell data. The development of minimum information about stem cell experiments (MIASCE) can further promote intelligent data aggregation and sharing.^58^ Significant progress has been made in our understanding of effective approaches for supporting shared, annotated stem cell research data.^59^,^60^ Strategies to optimize information technology supporting stem cell research and practice are being actively discussed and introduced into practice.^61^ Recognizing a crucial importance of sustainable information architecture for harmonized and intelligent data aggregation and sharing, the National Institute of Heart and Lung Disease (NHLBI) introduced recently the BioData Catalyst Strategic Framework that provides a forward-looking digital landscape of technologies, science and data.^62^ The BioData Catalyst is a cloud-based digital platform allowing easily find, access, share, store, cross-link, and compute on large-scale data sets. Innovative programs supporting intelligent stem cell data aggregation and sharing utilizing this platform are under way.^63^ Further expansion of intelligent bioinformatics solutions^64^ in stem cell research will provide new opportunities for analysis, evaluation and practical implementation of innovative stem cell technologies.
